# The Future of Insomnia Research—There's Still Work to Be Done

**DOI:** 10.1111/jsr.70091

**Published:** 2025-05-09

**Authors:** Raphael J. Dressle, Kai Spiegelhalder, Julian E. Schiel, Fee Benz, Anna Johann, Bernd Feige, Susanna Jernelöv, Michael Perlis, Dieter Riemann

**Affiliations:** ^1^ Department of Psychiatry and Psychotherapy Medical Center—University of Freiburg, Faculty of Medicine, University of Freiburg Freiburg Germany; ^2^ Institute of Medical Psychology and Medical Sociology Faculty of Medicine, University of Freiburg Freiburg Germany; ^3^ Division of Psychology Department of Clinical Neuroscience, Karolinska Institutet Stockholm Sweden; ^4^ Centre for Psychiatry Research Department of Clinical Neuroscience, Karolinska Institutet, Stockholm, Sweden and Stockholm Health Care Services Stockholm Sweden; ^5^ Department of Psychiatry University of Pennsylvania Philadelphia Pennsylvania USA

**Keywords:** CBT‐I, diagnosis, insomnia, insomnia models, neuroimaging, sleep

## Abstract

Insomnia Disorder (ID) is a highly debilitating disorder affecting up to 10% of the general population. In recent years, the number of studies in this area has increased rapidly, resulting in a wealth of accumulated knowledge. ID is generally regarded as a hyperarousal disorder affecting cognitive, emotional, cortical and physiological domains. Nevertheless, there is still a significant lack of knowledge about the pathophysiology of ID. For example, the existence of insomnia subtypes is discussed, albeit no uniform definition has yet been found. Significant progress has been made in understanding the neurobiology of insomnia, which points to a dysfunction in emotion regulation. However, neuroimaging studies frequently have small sample sizes and allow only for limited causal conclusions. The assessment of sleep has been significantly influenced by the increasing availability of methods for ambulatory sleep measurement. While these methods enable sleep to be measured more cost‐effectively than polysomnography, many devices lack sufficient empirical evidence of validity. In terms of insomnia treatment, cognitive behavioural therapy for insomnia (CBT‐I) has been shown to be highly effective. However, the underlying mechanisms of CBT‐I remain partially unclear, and the optimal sequence for applying the individual components, as well as the effectiveness of CBT‐I in cases of comorbidity, remain open questions. Furthermore, many widely applied pharmacological treatment approaches are used off‐label with only a limited empirical evidence base. This narrative review aims to summarise the current state of research on ID and attempts to outline a selection of the important future challenges in insomnia research.

## 1 State of the Science

According to the fifth edition of the Diagnostic and Statistical Manual of Mental Disorders (DSM‐5; American Psychiatric Association 2013), insomnia disorder (ID) is defined as difficulties falling asleep, maintaining sleep and/or waking up too early. These difficulties have to occur on at least three nights per week over a period of at least three months and need to be associated with an impairment of daytime functioning. Insomnia is a common disorder with prevalence estimates for an insomnia diagnosis ranging from 5.7% to 23.1% in European countries (Baglioni et al. 2020). Besides that, the economic and societal costs of insomnia are high. Wickwire et al. (2019) analysed the healthcare costs of untreated insomnia in the United States (US) and reported an increase of $63,607 in individual expenses over 11 months compared to individuals without insomnia, along with a strongly increased overall healthcare utilisation. In addition, insomnia generates substantial societal costs, for example, through reduced work productivity and absenteeism. Kessler et al. (2011) estimated these costs to surpass $63 billion annually in the US. When combining these different cost sources, annual costs of more than $100 billion dollars are estimated for the US (Wickwire et al. 2016).

In line with the high prevalence and economic consequences of insomnia, research on the disorder is booming, with a strong increase in publications during the past years. A simple PubMed search for the term “insomnia” results in 3650 hits for the year 2024. Ten years earlier, in 2014, only 1506 studies were published on insomnia, which equals an increase of 142% in this period. This is substantially higher than, for example, the increase in studies on “anxiety” (109%) and “depression” (69%) in the same period.

### 1.1 Aetiology of Insomnia

According to the 3P model of Spielman et al., the aetiology of ID can be described in terms of *predisposing*, *precipitating* and *perpetuating* factors (Riemann et al. 2022; Spielman et al. 1987). Predisposing factors relevant to the development of insomnia comprise a genetic predisposition (Jansen et al. 2019; Lane et al. 2019; Watanabe et al. 2022), personality traits like neuroticism or perfectionism (Dekker et al. 2017; Ellis et al. 2021; Fernandez‐Mendoza et al. 2010), a tendency to suppress aversive emotions (Kales et al. 1976; Park et al. 2012; van de Laar et al. 2010), and a disposition towards high cognitive and physiological arousability (Fernandez‐Mendoza et al. 2010). The onset of acute insomnia is mostly related to stressful life events that result in arousal levels which are capable of overcoming homeostatic sleep pressure (Riemann et al. 2022; Saper et al. 2005). Key factors that contribute to the perpetuation of insomnia symptoms and thus to the development of ID include maladaptive cognitions and behaviours, such as catastrophizing, prolonged nocturnal bedtimes, or daytime napping (Ellis et al. 2021; Morin 1993; Riemann et al. 2022).

### 1.2 Pathophysiology and Insomnia Models

Various models have been formulated that deal primarily with the maintenance of insomnia. The cognitive model of insomnia (Harvey 2002, 2005) highlights increased worry about the sleep disturbance and the consequences of poor sleep as a crucial factor, leading to both physiological arousal and anxiety. This then leads to increased selective attention towards threats for sleep, which ultimately results in a vicious circle: The likelihood that threats for sleep are detected is increased, which further increases worry. Empirically, Ellis et al. (2021) studied the transition from acute to chronic insomnia in a sample of 140 participants with acute insomnia. Their findings indeed highlight that affective sleep preoccupation, i.e., a tendency towards excessive worry and rumination about the sleep disturbance, serves as an important predictor for the persistence of insomnia symptoms (Ellis et al. 2021).

Espie et al. (2006) provided an elaborate description of specific maladaptive cognitive and behavioural processes associated with ID in the attention‐intention‐effort (AIE) model. The model suggests that insomnia is characterised by a disruption of the automaticity that sleep has in good sleepers. This is thought to be the result of selective *attention* to sleep, an explicit (mental) *intention* to sleep, and cognitive and behavioural sleep *effort*, e.g., attempts to suppress thoughts or use relaxation techniques in bed (Espie et al. 2006).

The neurocognitive model of insomnia (Perlis et al. 1997) emphasises the role of cortical arousal for the development and maintenance of insomnia. Cortical arousal is defined as an increase in high‐frequency EEG activity (in the beta and/or gamma range) and is thought to lead to an enhancement of information and sensory processing, particularly in the pre‐sleep period. This has also been linked to the phenomenon that many patients with insomnia show a significant underestimation of their objective polysomnography‐derived sleep duration (*subjective‐objective sleep discrepancy* or *paradoxical insomnia*, see Harvey and Tang 2012).

**FIGURE 1:**
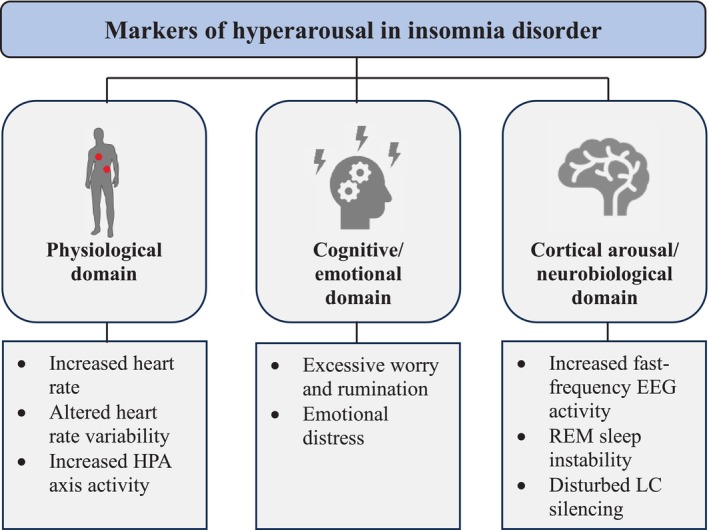
Markers of hyperarousal in patients with insomnia disorder. HPA = hypothalamic pituitary–adrenal; EEG = electroencephalography; REM = rapid eye movement; LC = locus coeruleus.

It is a unifying element of these insomnia models that they postulate some form of increased arousal that interferes with sleep, i.e., a process commonly associated with de‐arousal. This idea of being too aroused to sleep, of an “arousal mismatch”, has a long tradition in the scientific literature on insomnia. An important achievement of the last decades of insomnia research was that researchers were increasingly able to identify specific markers of hyperarousal in the physiological, cognitive/emotional and cortical domains (Dressle and Riemann 2023; Riemann et al. 2010, 2015; see Figure 1). Signs of physiological hyperarousal include findings demonstrating increases in heart rate and changes in heart rate variability (HRV) indicative of increased sympathetic activity (Bonnet and Arand 1998; Farina et al. 2014; Jarrin et al. 2016; Spiegelhalder et al. 2011), and increases in cortisol levels as markers of increased hypothalamic–pituitary–adrenal (HPA) axis activity (Dressle et al. 2022). An increase in high‐frequency electroencephalography (EEG) activity in patients as a sign of cortical arousal has been supported both meta‐analytically (Zhao et al. 2021) and by a recent large case–control study including 109 patients with ID and 109 age‐ and gender‐matched healthy controls (Dressle et al. 2023). With regard to cognitive/emotional hyperarousal, a large amount of research exists demonstrating increases in worry and rumination during the pre‐sleep period (Dressle et al. 2023; Hertenstein et al. 2015; Kalmbach et al. 2020; Nicassio et al. 1985; Palagini et al. 2016, 2017; Robertson et al. 2007; Vochem et al. 2019).

All of these hyperarousal processes may also underlie the increase in (micro‐)arousals during rapid eye movement (REM) sleep, which has been identified as another important pathophysiological phenomenon in patients with ID (i.e., *REM sleep instability* or *REM fragmentation*; Feige et al. 2023; Riemann et al. 2012, 2025). On the neurobiological level, REM sleep instability might be paralleled by a dysfunction of the locus coeruleus (LC), one of the core arousal‐promoting brain areas (Van Someren 2021; Wassing, Benjamins, et al. 2019, Wassing, Lakbila‐Kamal, et al. 2019, Wassing, Schalkwijk, et al. 2019). In healthy sleepers, firing of LC neurons is reduced during NREM sleep compared to wakefulness and hardly absent during REM sleep (Aston‐Jones and Bloom 1981). It is postulated that in patients with ID, silencing of LC neurons may be impaired during REM sleep, leading to a disturbance of synaptic plasticity processes that depend on low levels of norepinephrine. This could lead to a long‐term maintenance of emotional memory traces that are not disassociated from the limbic system, possibly resulting in an accumulation of distress and thereby increasing the risk for the development of comorbid mental disorders (Van Someren 2021; Wassing, Schalkwijk, et al. 2019). In addition, a study by Cano et al. (2008) suggests a key role for hyperarousal during sleep in patients with ID. To experimentally induce stress, the authors placed rats in either a clean or a dirty cage. The rats in the dirty cages began to develop sleep patterns similar to those of ID patients. For example, they showed an increase in sleep latency and sleep fragmentation. At the level of brain activity, a parallel activity was observed in arousal systems and sleep‐promoting systems, leading to the hypothesis that the homeostatic and circadian sleep pressure is not compromised in ID patients, while there is a parallel activity in wake‐promoting systems due to excitation by the limbic system (Cano et al. 2008).

### 1.3 Treatment Options

In current clinical guidelines, there is broad consensus that cognitive behavioural therapy for insomnia (CBT‐I) is recommended as first‐line treatment for ID (Morin et al. 2024; Qaseem et al. 2016; Ree et al. 2017; Riemann et al. 2023). CBT‐I is a multicomponent therapy that typically includes sleep restriction therapy (i.e., temporally reducing the time in bed to increase sleep pressure) and/or stimulus control therapy, the latter of which aims to strengthen the learned association between the bed environment and sleep. In addition, cognitive therapy targeting dysfunctional beliefs about sleep and sleeplessness is usually applied, along with sleep education and relaxation therapy (Espie 2022; Riemann et al. 2017). Overall, CBT‐I has considerable short‐term (van Straten et al. 2018) and long‐term effects (van der Zweerde et al. 2019).

Two recent network meta‐analyses focused on the relative efficacy of the individual CBT‐I components and found that sleep restriction therapy, stimulus control therapy and cognitive therapy are the key components of CBT‐I (Furukawa et al. 2024; Steinmetz et al. 2024). In this context, sleep restriction therapy (Kyle et al. 2023), stimulus control (Jansson‐Fröjmark et al. 2024; Verreault et al. 2024) and cognitive techniques (Jansson‐Fröjmark and Norell‐Clarke 2018) have also been shown to be effective as single‐component interventions. At the same time, relaxation techniques that are also frequently included in CBT‐I protocols were found to have potential detrimental effects on treatment outcome (Furukawa et al. 2024). Besides that, there is increasing evidence to support the efficacy of digitally delivered CBT‐I (Hasan et al. 2022; Simon et al. 2023).

Evidence‐based pharmacotherapeutic treatments for insomnia include benzodiazepines (BZ) and benzodiazepine receptor agonists (BZRA), dual orexin receptor antagonists, off‐label use of low‐dose sedative antidepressants, and prolonged‐release melatonin (Riemann et al. 2023). However, guidelines recommend offering pharmacotherapy only in cases in which CBT‐I is not sufficiently effective (Riemann et al. 2023).

## 2 Future Challenges

Despite this rapidly increasing knowledge, many relevant questions about insomnia remain unanswered. A selection of these will be discussed in the following sections.

### 2.1 Methodological Aspects of Insomnia Research

As in other areas of medical and psychological research, many results in insomnia research have not been adequately replicated. For example, one of the most influential neuroimaging studies on ID (Nofzinger et al. 2004) demonstrated a smaller decline in cerebral glucose metabolism from wake to NREM sleep in the patients group compared to a control group. This finding attracted a great deal of attention and contributed significantly to the model of hyperarousal. Nevertheless, it has only been partially replicated by the same research group (Kay et al. 2016, 2017), but not independently by others. Much more emphasis must be placed on replicating the most important findings of insomnia research. In this context, it may be beneficial when different research groups collaborate to mutually replicate their results (see, e.g., Schooler 2014).

In addition, there are various guidelines that formulate standards for conducting and reporting studies, e.g., the guidelines of the International Committee of Medical Journal Editors (ICMJE; https://www.icmje.org/) or the Consolidated Standards of Reporting Trials (CONSORT) statement (Boutron et al. 2017). Key principles include open science principles like the preregistration of studies and data sharing. Although it is difficult to assess comprehensively the extent to which these principles are currently being followed in insomnia research, we are convinced that there is scope for placing greater emphasis on them in future studies.

With regard to commonly applied study designs in insomnia research, the question of adequate control conditions appears essential. Future studies on insomnia treatment should also consider direct comparisons between CBT‐I and pharmacotherapy or between different pharmacotherapies. Only a small number of such studies exist, and the scarcity of comparative studies currently limits the evidence base for guideline recommendations and leads to great uncertainty about the optimal long‐term treatment of ID. In particular, it seems that the pharmaceutical industry has not been and most likely will not be incentivised to conduct comparative studies of hypnotic agents with CBT‐I or other hypnotic agents. Furthermore, there is currently no standard for what results should be reported in studies on CBT‐I, and there is a need to standardise a method for measuring adherence to CBT‐I (Muench et al. 2022).

### 2.2 Diagnosis of Insomnia

For decades, overnight polysomnography has been the primary diagnostic tool in sleep medicine. While polysomnography has provided valuable insight into basic functions of sleep as well as into numerous sleep disorders, it has two significant limitations, which have gradually reduced its use in routine clinical practice (Hirshkowitz 2016). First, many individuals sleep worse in the sleep laboratory than at home (Hu et al. 2024). This effect, probably caused by the discomfort of the electrodes, the unfamiliar environment, and the psychological consequences of the diagnostic evaluation (Hirscher et al. 2015), calls the validity of the assessment into question. Second, polysomnography in the sleep laboratory is rather expensive, limiting its use over multiple nights. However, day‐to‐day variability of sleep parameters across weeks or months is rather the norm than the exception (Chouraki et al. 2023) and may be an important predictor of cardiovascular and mental health (Zhu et al. 2022).

Because of these limitations, ambulatory devices have been developed to monitor sleep on a day‐to‐day basis at home (Kwon et al. 2021). These devices most often rely on the assessment of body movements and/or cardiorespiratory activity to monitor sleep in a manner that is less intrusive than in‐lab polysomnography. For some of these devices, a satisfactory validity has been demonstrated (e.g., Hamill et al. 2020; Kahawage et al. 2020). However, there is a large number of available commercial products that have never been validated following strict scientific standards. In addition, it is a major problem of this area of research and development that there is insufficient knowledge about the psychological aspects of sleep in those companies that develop ambulatory devices for measuring sleep. For example, an increased attentional focus on sleep is detrimental to sleep health (Espie et al. 2006), a phenomenon that is typically ignored by the developers of ambulatory devices and that warrants a clinically informed careful feedback of sleep parameters to device users. In this regard, it is still an open question what role objectively measured, quantitative criteria (e.g., > 30 min for sleep onset latency) should play in insomnia diagnosis or the assessment of severity.

### 2.3 Insomnia Models

#### 2.3.1 Are There Subtypes of ID?

The introduction of ID as an overarching diagnostic entity in the DSM‐5 has been celebrated as a milestone that has the potential to increase attention for insomnia from both a clinical and scientific perspective and to improve comparability between studies (e.g., Riemann et al. 2015). This represented a shift from an approach that focused on subtyping insomnia. For example, in the second version of the International Classification of Sleep Disorders (ICSD‐2; American Academy of Sleep Medicine 2005) the diagnosis of insomnia was divided into 11 categories. However, this (top‐down) subtype approach showed poor validity as a diagnostic strategy (Edinger et al. 2011).

In current pathophysiological research on insomnia, though, there are still significant approaches to subtyping insomnia. For example, Vgontzas et al. (2013) proposed two subtypes of ID, one with objective short sleep duration and a higher degree of physiological/somatic arousal and a second subtype with relatively normal sleep duration and a marked level of anxiety and cognitive arousal. Furthermore, there are data‐driven approaches that use a large amount of potentially relevant psychometric features to conduct a bottom‐up search for insomnia subtypes (Benjamins et al. 2017). In this context, Blanken et al. (2019) applied latent class analysis to questionnaire data from more than 2000 participants and identified five subtypes of insomnia patients (highly distressed, moderately distressed but reward sensitive, moderately distressed and reward insensitive, slightly distressed with high reactivity, slightly distressed with low reactivity).

These approaches have not yet led to a unified classification. Further research in this area seems clearly useful, not only to increase the precision of treatments in the future, but also to better reduce potential sources of heterogeneity in study samples.

#### 2.3.2 Do We Understand the Neurobiology of Insomnia?

As comprehensively summarised by Van Someren (2021), deviations in brain function related to the circadian or homeostatic components of sleep regulation appear to play a minor role in insomnia. Instead, impaired emotional reactivity and emotion regulation have emerged as important drivers of self‐perpetuating mechanisms in insomnia (Meneo et al. 2023) and central elements in recent neurobiological models of the disorder (Aquino and Schiel 2023). While aberrant emotional reactivity in insomnia is often conceptualised as increased stimulus‐related brain activation, emotion dysregulation is theorised to manifest as brain network hyperconnectivity.

Research on emotional reactivity in insomnia disorder (ID) has largely focused on the amygdala, a key structure in processing negative emotional stimuli. For instance, Baglioni and colleagues (Baglioni et al. 2014) used functional magnetic resonance imaging (fMRI) to assess amygdala reactivity (AR) to negative and neutral stimuli in ID. Their results suggested that patients with ID exhibit increased AR specifically in response to sleep‐related stimuli. However, findings remain inconsistent. Spiegelhalder et al. (2018) found no group differences when presenting sleep‐related words instead of pictures. The role of overnight emotional adaptation in ID was further examined by Wassing and colleagues, who demonstrated that disrupted REM sleep impairs the expected overnight reduction in AR (Wassing, Lakbila‐Kamal, et al. 2019). Their findings suggest that emotional reactivity in ID is not inherently heightened but insufficiently downregulated due to fragmented REM sleep. This aligns with broader models suggesting that REM sleep is crucial for emotional adaptation, a process that may be disrupted in ID (Feige et al. 2018; Riemann et al. 2012; Van Someren 2021). Corresponding assumptions, in turn, are based on animal studies that suggest a key role for REM sleep in memory consolidation (Li et al. 2017; Swift et al. 2018). As discussed above, this function of REM sleep appears to be closely linked to the silencing of the LC during this sleep phase. Swift et al. (2018) showed that optogenetic stimulation of the LC during sleep in rats impairs the consolidation of newly acquired spatial memories.

Functional connectivity magnetic resonance imaging (fcMRI) studies suggest that ID is associated with hyperconnectivity in key brain networks, particularly the default mode network (DMN), salience network (SN) and limbic system (LS; Khazaie et al. 2017; Schiel et al. 2020). The DMN, implicated in self‐referential thinking and rumination (Servaas et al. 2014), has shown altered connectivity patterns in ID, with hippocampal hyperconnectivity linked to sleep disturbances (Regen et al. 2016). However, between‐group differences in DMN connectivity remain inconsistent (Leerssen et al. 2019). The LS, particularly the anterior cingulate cortex and amygdala, has been implicated in persistent emotional distress in ID (Wassing, Lakbila‐Kamal, et al. 2019), suggesting impaired downregulation of negative affect over time. Similarly, the SN, which regulates stimulus detection and resource coordination, shows increased connectivity in ID (Chen et al. 2014). Together, these findings support a model where heightened emotional sensitivity, dysfunctional regulation and impaired neural downregulation perpetuate ID.

Despite these advances, major limitations persist in neuroimaging research on insomnia. Most studies use small samples and between‐group designs, limiting replicability (Reimann et al. 2023; Riemann et al. 2015; Tahmasian et al. 2018). Addressing these issues, large‐scale analyses have sought to clarify previous findings. For example, recent studies utilising data from the UK Biobank (UKBB) indicate that neither insomnia symptoms nor other sleep health variables show clear associations with AR, although chronic short sleep duration may be linked to blunted AR over time (Schiel et al. 2022). This suggests that differences in emotional reactivity may not be inherent to ID but could emerge as compensatory adaptations to (subjective) chronic sleep loss. Similarly, morphometric analyses indicate that structural brain alterations in ID are minimal (Schiel et al. 2023; Weihs et al. 2023), reinforcing the notion that functional rather than anatomical differences (e.g., hippocampal hyperconnectivity; Holub et al. 2023) underlie emotion dysregulation in the disorder (Aquino et al. 2024).

Given these complexities, our understanding of the neurobiology of insomnia remains incomplete. While significant progress has been made in linking ID to alterations in emotional reactivity and regulation, findings remain inconsistent and mechanistic clarity is still lacking. The transition from region‐based to network‐level analyses has been crucial for understanding emotion‐related dysfunction in ID. However, further methodological refinement is needed, balancing large‐scale epidemiology with targeted experiments to clarify the interplay between sleep and emotion in ID.

### 2.4 Insomnia Treatment

Although CBT‐I is highly effective, there are still many challenges with regard to insomnia treatment. These relate, for example, to the delivery of CBT‐I in online settings, further improvements to CBT‐I and the integration of new therapeutic approaches, the treatment of special groups of insomnia patients with CBT‐I (e.g., those with comorbid sleep apnea), and new forms of pharmacological treatment. Furthermore, it is unclear how many sessions are optimal to achieve specific results (Muench et al. 2022).

Internet‐delivered CBT‐I has been advocated as a convenient format of an evidence‐based treatment mainly due to its efficacy, cost‐effectiveness and accessibility. However, there is insufficient evidence on non‐inferiority to face‐to‐face CBT‐I as only a few studies have directly compared the two formats with each other (Blom et al. 2015b; Lancee et al. 2016; Taylor et al. 2017). Currently, a multicentre clinical trial in Germany is being conducted testing whether guided internet‐based CBT‐I is as effective as face‐to‐face CBT‐I in over 400 patients (Benz et al. 2024).

Although CBT‐I is highly effective and recommended as first‐line treatment, up to 60% of insomnia patients do not fully remit after CBT‐I (Morin et al. 2009). In fact, little is known about the mechanisms by which CBT‐I exerts its effects. The lack of mechanistic research is partly due to the methodological problem that CBT‐I is usually evaluated as a treatment package and the contribution of individual components is not systematically examined (Espie 2022; Parsons et al. 2021). It is likely that the components of the intervention achieve their effects through different mechanisms. For example, while the behavioural components of CBT‐I may have a direct effect on physiologic sleep fragmentation by increasing homeostatic sleep pressure, cognitive therapy may primarily act by reducing worry and unhelpful beliefs (Harvey et al. 2017). One relatively consistent finding is that the effects of CBT‐I on sleep are primarily found in self‐report rather than in objective, polysomnographically defined measures of sleep (Mitchell et al. 2019). Research on other objective changes as a result of CBT‐I has yielded inconsistent results. For example, studies have not found a decrease in cortisol levels as a result of CBT‐I (Miller et al. 2015; Vgontzas et al. 2020). Regarding cortical arousal, Maurer et al. (2022) found a significant decrease in relative beta power after sleep restriction therapy compared to the control intervention. Nevertheless, the effect was only small and needs replication. Thus, there is a need for prospective studies designed to focus on the investigation of treatment mechanisms. Understanding the psychobiological mechanisms of the different CBT‐I components may help to refine and improve the first‐line treatment for patients with insomnia. By identifying biomarkers or patient phenotypes most likely to respond, the therapy could also become more personalised to the individual patient's needs, for example, by incorporating additional therapeutic elements that target specific mechanisms. If therapy mechanisms are linked to particular biological or neurological pathways, pharmaceutical interventions could be developed to mimic or enhance its effects.

The ideal sequence in which CBT‐I components are delivered is also an open question. One may argue that sleep restriction therapy has the strongest evidence base (Furukawa et al. 2024; Steinmetz et al. 2024), and thus should be administered first. However, others may argue that a therapeutic alliance should be established before delivering the behavioural components of CBT‐I since these can be quite demanding and can have considerable side effects (Kyle et al. 2014). Hence, future studies could test different sequences of techniques within CBT‐I in terms of effectiveness and dropout rates. In this context, personalising treatments could also be important, as different sequences may be appropriate depending on the patient.

Since, as already discussed, a considerable proportion of patients do not show full remission following CBT‐I treatment (Morin et al. 2009), current research started to investigate whether concepts or techniques from other treatment approaches might be added to the existing CBT‐I protocol in order to increase treatment response. Acceptance and Commitment Therapy (ACT), for example, is a therapy approach that has emerged from the third wave of cognitive‐behavioural therapy. It focuses on helping patients to stay in the present moment and to live a value‐based life without judging feelings and behaviour (Hayes et al. 2012). With regard to insomnia, ACT might help patients to shift their attention away from sleep and sleep problems, which contribute to maintaining the sleep problem. ACT might also help insomnia patients to live their lives in line with their personal values. This may support patients to cope better with the side effects of CBT‐I (Hertenstein et al. 2024). A systematic review that investigated the effect of ACT on insomnia and sleep quality showed mixed results and considerable methodological variation (Salari et al. 2020). A recent clinical trial found that ACT combined with sleep restriction therapy had a positive effect on a number of health outcomes, e.g., energy level, compared to stand‐alone CBT‐I (Hertenstein et al. 2024), and a recent study on ACT alone compared to CBT‐I showed that ACT had an effect also when not combined with sleep restriction therapy, albeit delayed (El Rafihi‐Ferreira et al. 2024). As such, ACT may have the potential to serve as an add‐on to CBT‐I, helping manage side effects and thus fostering patient adherence.

In addition, it may be reasonable to allocate more resources to clinical trials that investigate the efficacy of CBT‐I in patients with specific comorbid conditions when there is a strong rationale for why these comorbid conditions may limit CBT‐I efficacy or require an adaptation of the treatment. For example, a recent meta‐analysis has summarised clinical trials on the efficacy of CBT‐I in the treatment of patients with comorbid insomnia and sleep apnoea (COMISA; Sweetman et al. 2023). According to this analysis, there is compelling evidence that CBT‐I improves insomnia‐related outcomes in this group of patients; however, findings on OSA‐related outcomes are more heterogeneous. A further research question that is highly relevant from a clinical point of view and clearly needs further scientific attention is the question of the optimal sequencing of CBT‐I and positive airway pressure (PAP) therapy in patients with COMISA. There is some moderate‐level evidence suggesting that OSA treatment improves insomnia severity (Sweetman et al. 2021a). This supports the assumption that insomnia symptoms can be secondary to OSA in some patients and that OSA treatment should be initiated first before insomnia treatment. However, following the logic that insomnia is a significant barrier to PAP adherence, one could also argue that CBT‐I should be conducted before PAP initiation. Unfortunately, there is little evidence available to guide clinicians with respect to the question of sequencing (Ong et al. 2020). Regarding a potential adaptation of CBT‐I for patients with COMISA, it has to be considered that these patients report more pronounced daytime sleepiness compared to patients with insomnia alone (Sweetman et al. 2021b) and increased sleepiness is a common transient side effect of sleep restriction therapy (Kyle et al. 2014). Thus, it seems reasonable to monitor patients' levels of sleepiness more rigorously in this group. In addition, a minimum recommended time in bed of 6 h is often used, which is substantially higher than the 4½–5 h that are typically recommended for insomnia patients without COMISA.

With regard to pharmacotherapy, there is a large discrepancy between the prescription frequency of commonly used substances and scientific evidence. For example, sedating antidepressants are commonly used in Europe to treat insomnia. Nevertheless, none of these drugs are officially approved for insomnia without comorbid depression, making off‐label use the norm (Riemann et al. 2023). A meta‐analysis conducted by Everitt et al. (2018) indicates that evidence supporting the use of sedating antidepressants for treating insomnia is limited. They found only a few and mostly small studies with design limitations and short‐term follow‐up. Thus, high‐quality randomised controlled trials and long‐term studies on pharmacological treatments for insomnia are needed.

#### 2.4.1 Benefits of Incorporating CBT‐I Into Treatment of Other Psychiatric Disorders

It has been convincingly demonstrated that CBT‐I has positive effects on secondary outcomes that are related to psychopathology in general (e.g., depression or anxiety symptoms; Benz et al. 2020). In addition, a meta‐analysis by Hertenstein et al. (2022) demonstrated that CBT‐I is generally effective in patients with comorbid insomnia and depression, PTSD and alcohol dependency, both regarding insomnia and comorbid symptom severity. However, many of the included primary studies compared CBT‐I alone with a placebo condition or sleep education, making it still rather unclear if the inclusion of CBT‐I in routine care for mental disorders has beneficial effects in comparison to standard guideline treatment. In addition, there is a lack of research on other groups of diagnoses, such as attention deficit hyperactivity disorder (ADHD), eating disorders, somatoform disorders and personality disorders (Hertenstein et al. 2022), and there is a lack of studies investigating the effects of CBT‐I delivered by ordinary health care staff in regular clinical practice.

Simultaneously, it is a common belief among psychiatrists that insomnia is a symptom that manifests most frequently in patients with anxiety disorders and unipolar depression. Indeed, 60%–90% of the patients affected by these mental disorders also suffer from insomnia (Papadimitriou and Linkowski 2005; Seow et al. 2016). The perception of insomnia as a symptom goes along with the view that treatment should focus on the “underlying” disorder and that effective anxiety or depression treatment also results in a sufficient improvement of insomnia. However, the scientific evidence for this view is comparably weak. Blom and colleagues (Blom et al. 2015a) demonstrated in a sample of patients with depression and ID that treatment of insomnia with CBT‐I alleviated both insomnia and depression, whereas treatment with CBT for depression was less effective in alleviating insomnia symptoms. In addition, a recent study by the same group found that a combination of CBT‐I and CBT for depression does not outperform CBT‐I alone to treat depression in a sample with comorbid ID and depression (Blom et al. 2024).

## 3 Suggestions for Future (Research) Practice

Based on the current state of research and the emerging trends in insomnia research discussed above, we consider the following aspects to be central to future research and clinical practice:

1. Researchers in the field should put more effort into replicating important findings of insomnia research. Furthermore, increasing adherence to open science principles will further improve research.
2. The number of ambulatory sleep assessment tools is rapidly increasing. It seems crucial to not only investigate the validity of specific tools, but also the general effects of their use on insomnia patients. This could also help to formulate more evidence‐based recommendations for diagnosis that go beyond those that are usually based on clinical experience.
3. Data‐driven approaches appear to be useful to gain a deeper understanding of possible subtypes of insomnia. Such subtyping, which is also conceivable using other approaches, offers the advantage of potentially better personalisation of therapeutic approaches. It could also reduce study sample heterogeneity in studies that aim to obtain homogeneous samples.
4. It seems important to study the effects, benefits and difficulties of delivering CBT‐I in sub‐groups such as different psychiatric disorders, and whether adaptations may be needed for different subgroups of patients.
5. Future research should include approaches to outcome prediction to personalise treatment, as well as the use of adaptive treatment strategies to accommodate specific patient needs that were not possible to identify before treatment started.
6. Dissemination and implementation are still under‐investigated areas. Stepped care approaches need to be developed and studied, as well as strategies to strengthen implementation and maintenance.
7. Our understanding regarding the neurobiology of insomnia is incomplete. Findings on changes in emotional reactivity and regulation, among other things, are inconsistent and there is still a lack of mechanistic clarity. The frequently small sample sizes represent a crucial problem. Future research in this area should therefore include both large‐scale epidemiological studies and targeted experimental manipulations.
8. With regard to insomnia treatment, there is a significant need for a better understanding of CBT‐I mechanisms. In addition, the optimal administration sequence of different CBT‐I components should be empirically investigated. Furthermore, it may be useful to conduct more studies on the efficacy of CBT‐I in patients with specific comorbidities (e.g., sleep apnea) and on the efficacy of CBT‐I as an add‐on treatment for mental disorders. With regard to pharmacological treatment approaches, the off‐label treatment of sleep disorders, e.g., with sedative antidepressants, is widespread. In this context, too, more high‐quality randomised controlled trials are needed.
9. An overarching question is how all these empirical findings can be communicated to the public. It is a hallmark of ID that affected patients tend to ruminate about their sleep disturbances and their potential (health) consequences (e.g., Harvey and Greenall 2003). Indeed, research findings suggesting adverse health consequences, such as increased risk of cardiovascular disease and hypertension (Benz et al. 2023) appear to support these concerns, although it is likely that the magnitude of risk is commonly overestimated. Nevertheless, a dilemma arises: how should researchers studying insomnia communicate about ID and relevant findings without further fueling concerns and thereby exacerbating the problem at the societal level? (see Fernandez‐Mendoza 2018, for a similar discussion).

## Author Contributions

**Raphael J. Dressle:** conceptualization, writing – original draft, writing – review and editing. **Kai Spiegelhalder:** conceptualization, writing – original draft, writing – review and editing. **Julian E. Schiel:** writing – original draft, writing – review and editing. **Fee Benz:** writing – original draft, writing – review and editing. **Anna Johann:** writing – original draft, writing – review and editing. **Bernd Feige:** writing – review and editing. **Susanna Jernelöv:** writing – review and editing. **Michael Perlis:** writing – review and editing. **Dieter Riemann:** conceptualization, writing – original draft, writing – review and editing.

## Conflicts of Interest

Raphael J. Dressle, Kai Spiegelhalder, Julian E. Schiel, Fee Benz, Anna Johann, Bernd Feige, Susanna Jernelöv, and Michael Perlis do not report any financial or non‐financial conflicts of interest. Dieter Riemann has received honoraria for speaking engagements from GET.ON Institut für Online Gesundheitstrainings GmbH, Heel and Idorsia. Dieter Riemann has received consultancy fees from GAIA.AG, Heel, Idorsia and X‐trodes.

## Data Availability

Data sharing is not applicable to this article as no new data were created or analyzed in this study.
